# Automatic 3D Surface Reconstruction of the Left Atrium From Clinically Mapped Point Clouds Using Convolutional Neural Networks

**DOI:** 10.3389/fphys.2022.880260

**Published:** 2022-04-27

**Authors:** Zhaohan Xiong, Martin K. Stiles, Yan Yao, Rui Shi, Aaqel Nalar, Josh Hawson, Geoffrey Lee, Jichao Zhao

**Affiliations:** ^1^ Auckland Bioengineering Institute, The University of Auckland, Auckland, New Zealand; ^2^ Waikato Clinical School, Faculty of Medical and Health Sciences, The University of Auckland, Auckland, New Zealand; ^3^ Fuwai Hospital, Beijing, China; ^4^ Royal Melbourne Hospital, Melbourne, VIC, Australia

**Keywords:** convolutional neural network, left atrium, point cloud, sparse data, 3D surface reconstruction

## Abstract

Point clouds are a widely used format for storing information in a memory-efficient and easily manipulatable representation. However, research in the application of point cloud mapping and subsequent organ reconstruction with deep learning, is limited. In particular, current methods for left atrium (LA) visualization using point clouds recorded from clinical mapping during cardiac ablation are proprietary and remain difficult to validate. Many clinics rely on additional imaging such as MRIs/CTs to improve the accuracy of LA mapping. In this study, for the first time, we proposed a novel deep learning framework for the automatic 3D surface reconstruction of the LA directly from point clouds acquired *via* widely used clinical mapping systems. The backbone of our framework consists of a 30-layer 3D fully convolutional neural network (CNN). The architecture contains skip connections that perform multi-resolution processing to maximize information extraction from the point clouds and ensure a high-resolution prediction by combining features at different receptive levels. We used large kernels with increased receptive fields to address the sparsity of the point clouds. Residual blocks and activation normalization were further implemented to improve the feature learning on sparse inputs. By utilizing a light-weight design with low-depth layers, our CNN took approximately 10 s per patient. Independent testing on two cross-modality clinical datasets showed excellent dice scores of 93% and surface-to-surface distances below 1 pixel. Overall, our study may provide a more efficient, cost-effective 3D LA reconstruction approach during ablation procedures, and potentially lead to improved treatment of cardiac diseases.

## 1 Introduction

Point clouds are a widely used method of storing information acquired in the ever-growing world of data (Rusu et al., 2008; Guo et al., 2020). Current advancements in 3D acquisition technology in the form of sensors, scanners, and imaging capture high-quality data to allow for more refined research of their components and properties (Pomerleau et al., 2015). In particular, the acquisition of 3D data in the medical field is an increasingly important area of study in terms of visualizing organ structures, recording real-time anatomical information during surgery, and physiological mapping (Ptaszek et al., 2018; Kim et al., 2020). Compared to 3D imaging, point clouds are significantly more memory-efficient by storing information in a compact and vectorized form. This data format also enables efficient manipulation using simple mathematical operations with low computational costs.

In recent years, medical recording technology, particularly devices in cardiology, has integrated point clouds into the systems for various applications. Catheter ablation is one of the most common clinical procedures for treating complex cardiac diseases such as arrhythmia. During the procedure, an estimated geometry of the cardiac chamber is initially constructed using point-by-point catheter recordings on the endocardial surface (Rolf et al., 2014). The geometry formed from the point cloud is then used to guide and target specific regions containing diseased heart tissue for ablation (Hansen et al., 2015). Therefore, accurate reconstruction of cardiac chambers from point clouds is vitally important for the effectiveness of the procedure. This is especially the case for atrial chamber reconstruction during catheter ablation of atrial fibrillation, the most common cardiac arrhythmia (Xiong et al., 2018; Xiong et al., 2021).

Current methods of point cloud to atrial chamber reconstruction, particularly left atrium (LA), are heavily commercialized and not openly accessible. The two most widely used commercial anatomical mapping systems are the EnsiteNavX (St Jude Medical, Minnesota, United States) and CARTO 3 (Biosense Webster, California, United States). To ensure accurate LA models are produced, clinicians further merge the point cloud with anatomical LA segmentations obtained from magnetic resonance imaging (MRI) or computed tomography (CT) in advance of the procedure. There is limited research aiming to improve the efficiency and accuracy of LA reconstruction algorithms. The only notable study is Baram et al. who proposed an auto-encoder to perform LA reconstruction from simulated catheter points and LA geometries (Baram et al., 2018). The methods proposed were not tested directly on real data and lacked rigorous validation. Therefore, there is a need for a more accurate and robust algorithm capable of fully automatic LA reconstruction directly from point clouds.

Convolutional neural networks (CNNs) are currently the main driver of modern analytical methods for structured data (Zhang et al., 2019). The major differences when implementing CNNs for point clouds as opposed to traditional pixels or voxels are the variable lengths and unordered structure of point cloud vectors. This has led many studies to design specialized approaches that adapt CNNs to their respective task, as they have already been proven to be extremely robust in imaging analysis (Ronneberger et al., 2015; Milletari et al., 2016). As the point cloud data is required to be standardized into a consistent shape for the CNN, approaches mainly focus on normalizing the data with pre-processing. Projection-based methods involve mapping 3D point clouds onto 2D surfaces at different angles (Yu et al., 2018), or onto standardized spherical representations (Lawin et al., 2017), which can be then analyzed directly. These studies have focused on selecting the best projection approach, such as using CNNs to analyze multiple projections of the same set of points and aggregating the results to obtain a more robust prediction (Audebert et al., 2016). Some studies also use CNNs to perform predictions on projections of local points due to the more consistent geometry in a regional area, followed by aggregation of the local outputs into a global prediction (Tatarchenko et al., 2018). Spherical projections have been more commonly used as more information can be retained in a single 2D representation, although this results in a loss of local details (Milioto et al., 2019). A more straightforward method is discretization, in which the 3D point clouds are converted into volumetric images which can be directly analyzed by CNN (Tchapmi et al., 2017). Studies have investigated ways to optimize methods of discretization due to the computationally expensive nature of this type of volume-based analysis. Some approaches have partitioned point clouds into a lattice of voxels, in which each voxel is processed differently depending on the number of points present (Meng et al., 2019). To improve accuracy, studies have used adaptive voxel sizes to target regions of high point density and ignore low-density regions (Graham et al., 2018). This increases the resolution of the discretized representation of the point set in the regions containing interest without increasing the computational burden.

The recent advancements in CNNs for point cloud analysis have provided a solid baseline for developing a LA point cloud analysis approach. Despite these studies, there still lacks research progress for converting sparse point clouds to volumetric geometries, especially in the medical field. Potential solutions for this complex task could involve state-of-the-art CNNs for 3D medical image segmentation, which specialize in the image reconstruction of extremely fine structures (Ronneberger et al., 2015; Milletari et al., 2016). The popular 3D U-Net architecture (Ronneberger et al., 2015) has been implemented for a wide range of tasks including heart segmentation (Zhuang et al., 2019), and its enhanced version, V-Net (Milletari et al., 2016), achieves further performance improvements. A recent global benchmarking study has also experimentally deduced the most optimal U-Net CNN configuration for LA segmentation from 3D MRIs (Xiong et al., 2021), surpassing traditional and other CNN methods. A 2019 benchmarking study for ventricular segmentation also demonstrated the highest-scoring team utilizing an enhanced U-Net approach (Wu et al., 2021). Furthermore, a recent review by Wu et al. outlined the advantages of CNNs, particularly those with U-Net backbones, over conventional atlas and registration-based methods for LA and scar segmentation (Yang et al., 2020). A Multi-view attention CNN was further developed to improve accuracies over standard CNNs (Kingma and Ba, 2014). Thus, we believe an approach which leverages both leading point cloud analytical techniques and medical imaging CNNs is the best strategy for tackling the task in this study.

In this study, we proposed the first deep learning pipeline for fully automatic surface reconstruction of the LA from point cloud data. Our method achieved anatomically accurate LA predictions directly from point clouds without the need for additional imaging. We tested the framework on independent clinical datasets acquired using the two most widely used commercial mapping systems. Our study may potentially be used to improve current mapping systems for guiding ablation procedures to treat cardiac diseases.

## 2 Methods

### 2.1 CNN for LA Reconstruction

**FIGURE 1:**
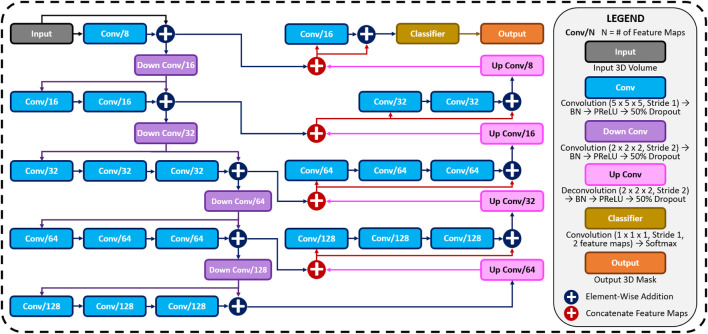
The architecture of the proposed 3D convolutional neural network (CNN) for predicting the left atrial (LA) geometry from a point cloud obtained during clinical mapping. The number of kernels in each convolutional layer is shown, along with the type of convolution. The flow of the gradients between layers is also shown, with different operations for merging two layers. The legend shows the exact operations of each layer labelled with different colors. All parameters can be found in Table 1. BN, batch normalization; conv, convolution; PReLU, parametric rectified linear unit.

A CNN was developed to predict the 3D surface LA geometry given the point-cloud recording of the LA during clinical mapping. The architecture is shown in Figure 1, and the full summaries of parameters are shown in Table 1. The point cloud was first pre-processed into a fixed input volume. All inputs were then cropped to a standard size of 128 × 208 × 88 pixels, removing background pixels to alleviate class imbalance. The CNN architecture consisted of a modified 3D U-Net architecture with additional residual connections to improve the convergence. We used a fully convolutional network to decrease computational costs and ensure the CNN operates independent of input size. The CNN was relatively light-weight as the maximum number of convolutional kernels per layer was 128. This further ensured faster training and convergence, as well as being significantly less memory intensive.

**TABLE 1:** The configurations of the convolutional neural network.

Encoder path layers	Kernel size	Stride	Feature maps	Number of parameters
Input 3D	-	-	1	-
Conv/8	5 × 5 × 5	1	8	5 × 5 × 5 × 1 × 8
Down Conv/16	2 × 2 × 2	2	16	5 × 5 × 5 × 8 × 8
Conv/16	5 × 5 × 5	1	16	5 × 5 × 5 × 16 × 16
Conv/16	5 × 5 × 5	1	16	5 × 5 × 5 × 16 × 32
Down Conv/32	2 × 2 × 2	2	32	5 × 5 × 5 × 32 × 32
Conv/32	5 × 5 × 5	1	32	5 × 5 × 5 × 32 × 32
Conv/32	5 × 5 × 5	1	32	5 × 5 × 5 × 32 × 32
Conv/32	5 × 5 × 5	1	32	2 × 2 × 2 × 32 × 64
Down Conv/64	2 × 2 × 2	2	64	5 × 5 × 5 × 64 × 64
Conv/64	5 × 5 × 5	1	64	5 × 5 × 5 × 64 × 64
Conv/64	5 × 5 × 5	1	64	5 × 5 × 5 × 64 × 64
Conv/64	5 × 5 × 5	1	64	2 × 2 × 2 × 64 × 128
Down Conv/128	2 × 2 × 2	2	128	5 × 5 × 5 × 128 × 128
Conv/128	5 × 5 × 5	1	128	5 × 5 × 5 × 128 × 128
Conv/128	5 × 5 × 5	1	128	5 × 5 × 5 × 128 × 128
Conv/128	5 × 5 × 5	1	128	5 × 5 × 5 × 128 × 128

Decoder path layers	Kernel size	Stride	Feature maps	Number of parameters
Up Conv/64	2 × 2 × 2	2	64	2 × 2 × 2 × 128 × 64
Conv/128	5 × 5 × 5	1	128	5 × 5 × 5 × 128 × 128
Conv/128	5 × 5 × 5	1	128	5 × 5 × 5 × 128 × 128
Conv/128	5 × 5 × 5	1	128	5 × 5 × 5 × 128 × 128
Up Conv/32	2 × 2 × 2	2	32	2 × 2 × 2 × 128 × 32
Conv/64	5 × 5 × 5	1	64	5 × 5 × 5 × 64 × 64
Conv/64	5 × 5 × 5	1	64	5 × 5 × 5 × 64 × 64
Conv/64	5 × 5 × 5	1	64	5 × 5 × 5 × 64 × 64
Up Conv/16	2 × 2 × 2	2	16	2 × 2 × 2 × 64 × 16
Conv/32	5 × 5 × 5	1	32	5 × 5 × 5 × 32 × 32
Conv/32	5 × 5 × 5	1	32	5 × 5 × 5 × 32 × 32
Up Conv/8	2 × 2 × 2	2	8	2 × 2 × 2 × 32 × 8
Conv/16	5 × 5 × 5	1	16	5 × 5 × 5 × 16 × 16
Classifier	1 × 1 × 1	1	2	1 × 1 × 1 × 16 × 2
Output 3D	-	-	2	-
		Total Parameters		~ 32.5 Million

Conv, convolution.

The first half of the CNN was an encoder to learn dense features from the input through several convolutional layers of increasing depth. The convolutional layers contained 5 × 5 × 5 kernels and a stride of 1 for an increased receptive field over traditional 3 × 3 × 3 kernels, and the number of feature maps increased from 8 to 128. At every 1 to 3 convolutional layers, residual connections were added to improve feature learning and 2 × 2 × 2 convolutions with a stride of 2 were used to progressively down-sample the input by a factor of 2. The additional residual connections did not contribute to an increase in parameters but greatly increased information flow throughout the network, allowing important features to be retained as the input is down-sampled. The use of convolutions to down-sample the input as opposed to traditional pooling also implicitly enabled the CNN to learn the important features while removing unimportant information during compression.

The second half of the CNN was a decoder used to reconstruct the input back to the original resolution through several 5 × 5 × 5 convolutional layers of decreasing depth. This was done to facilitate subsequent segmentation. The number of feature maps of the convolutions in this part of the network decreased from 64 to 16. The input was progressively up-sampled by a factor of 2 with 2 × 2 × 2 deconvolutional, or transpose convolutional, layers with stride of 2. Residual connections were added at every 1–3 convolutional layers. In order to directly preserve high-resolution features from the input, feature forwarding connections were also used to concatenate the outputs of the convolutional layers in the encoder path to those in the decoder path at four different points along the CNN. This allowed the CNN to learn from both raw high-level features as well as condensed low-level features. This also greatly improved the consistency of reconstruction by essentially guiding the output to be representative of the input information. Overall, apart from the final output layer, batch normalization and parametric rectified linear units (PReLU) were used after every convolutional layer along with the entire CNN for normalization, and 50% dropout was used at every layer for regularization to decrease overfitting. The final output layer of the CNN contained a 1 × 1 × 1 convolution with a stride of 1 and a softmax activation function to predict for zeros (background) and ones (LA pixel).

The hyper-parameters in the CNN were selected through controlled experimentation to determine the optimal configuration for the task. The number of convolutional kernels was tuned using 4, 8, and 16 kernels for the first layer, with the remaining layers doubling as described. Experiments showed that using four kernels did not provide the network with sufficient depth to predict the LA accurately while 16 kernels were too computationally intensive with minimal improvement over eight kernels. The number of steps in the encoder and decoder paths was also adjusted to find the degree of compression needed. Similar with the number of kernels, CNNs without sufficient down-sampling steps were too shallow for the task, while the number of down-sampling steps above the optimal four steps did not contribute to an increase in accuracy. We implemented a CNN with 3 × 3 × 3 kernels and compared the results with the 5 × 5 × 5 kernels. Surprisingly, the network had difficulty converging when using size 3 kernels, potentially due to the lack of receptive field which could not effectively process the sparse inputs provided. We found PReLU activations worked more harmoniously with the network architecture compared with ReLU and leaky ReLU as it produced the best performances. The percentage of the dropout was also tuned with dropout rates of 25%, 50%, and 75%. While the performance did not significantly vary, a drop out of 50% provided sufficient regularization without reducing the training time as when applying 75% dropout.

To alleviate class imbalance, a dice loss function was used during training to assign higher priorities to the pixels containing the atria during prediction. The dice loss also increased the speed of convergence, significantly reducing computational costs. The formulation of the dice loss, *F*_*dice*_(*p, g*), where *p* and *g* represents the predicted and ground truth 3D binary masks, was

$$F_{dice}\left(p,g\right)=\frac{2\sum_{x}\sum_{y}pg+1}{\sum_{x}p^{2}+\sum_{y}g^{2}+1}$$ (1)

where *p* and *g* were of dimensions of *x* and *y*.

The adaptive moment estimation (ADAM) gradient descent optimizer (McGann et al., 2014) was used to minimize the loss function during training with a constant learning rate of 0.0001 and the exponential decay rates of the 1st and 2nd moment estimates were set to 0.9 and 0.999, respectively. To reduce the computational burden of the large images that needed to be processed, all data was stored in the hierarchical data format after pre-processing. The CNN was trained with a maximum limit of 1,000 epochs, with a criterion to stop training if the accuracy on the validation set did not improve after 50 epochs. A batch size of 1 was used due to the high memory costs associated with 3D volumes. The training set was also shuffled for each epoch to increase randomness. After every epoch, the performance of the CNN was evaluated on the validation set with the dice score. The parameter set of the CNN which achieved the highest validation accuracy was saved and used on the testing set. The CNN was developed in TensorFlow, an open-source Python deep learning library, and TFLearn, a high-level Python API for Tensorflow. The training step was performed on an Nvidia Titan V GPU with 5120 CUDA cores and 12 GB RAM. The training phase took approximately 10 hours. Predictions took approximately 10 s for each partial shell input.

## 3 Experimental Setup

### 3.1 Data and Pre-Processing

A summary of the three datasets (*paired training data*, *test #1*, *test #2*) used in this study is shown in Table 2. The CNN was initially trained on a generated dataset (*paired training data*) and tested on two clinical datasets (*test #1* and *test #2*). The generated dataset was simulated to provide sufficient samples to train the CNN, as clinical data is time-consuming and expensive to acquire. The two clinical datasets both contained LA surface geometries segmented from MRIs or CTs and point clouds acquired with the most widely used commercial mapping systems merged into the same coordinates as the imaging. This provided matching pairs of input point clouds and output LA for testing the CNN. The following sub-sections describe the generation and acquisition of the three datasets in detail.

**TABLE 2:** Summary of the data used in this study.

Dataset	Training	Validation	Testing
*Paired training data*	1,000	240	300
*Test#1*: clinical MRI + point cloud	—	—	4
*Test#2*: clinical CT + point cloud	—	—	2

CT, computed tomography; MRI, magnetic resonance imaging.

The Waikato clinical study was approved by New Zealand Health and Disability Ethics Committees (Ref: 16/STH/130) and the ethics approval for the studies at other centers at Utah (Xiong et al., 2018; Yang et al., 2020), Beijing (Kingma and Ba, 2014) and Melbourne (Edelsbrunner and Mücke, 1994) were already obtained.

#### 3.1.1 Paired Training Data

The *paired training* dataset was generated by merging two separate datasets: 154 LA surface geometries manually segmented from MRIs (Yao et al., 2007) and 10 sets of point clouds of the LA recorded with clinical mapping (Edelsbrunner and Mücke, 1994). The point clouds were transformed to fit the same spatial coordinates as the LA segmentations, forming matching pairs of point cloud and LA geometries available for the CNN. Overall, 1,540 data samples were generated by exhausting all pairing combinations of the two datasets.

The 154 3D MRIs with a spatial resolution of 0.625 mm × 0.625 mm × 0.625 mm were acquired from patients with atrial fibrillation at the University of Utah, United States (Yao et al., 2007). The LA geometries were manually segmented in agreement with three expert observers for each scan. Segmentations were initially performed by one observer and modified by a second observer in agreement with the first observer to ensure accuracy and consistency. Where there was a disagreement between the first two observers, a third observer was consulted to mediate and further refined the segmentation. The LA was defined as the pixels contained within the LA endocardial surface, including the four pulmonary veins (PVs). The 3D coordinates of each PV in each LA were also recorded for landmark registration.

The 10 point cloud data were created with clinical mapping during catheter ablation to treat patients with atrial fibrillation in Beijing, China (Edelsbrunner and Mücke, 1994). Similar to the MRIs, the coordinates of the four PVs were annotated in the maps. The average number of coordinates recorded for the point clouds were 3,703 ± 1,043.

**FIGURE 2:**
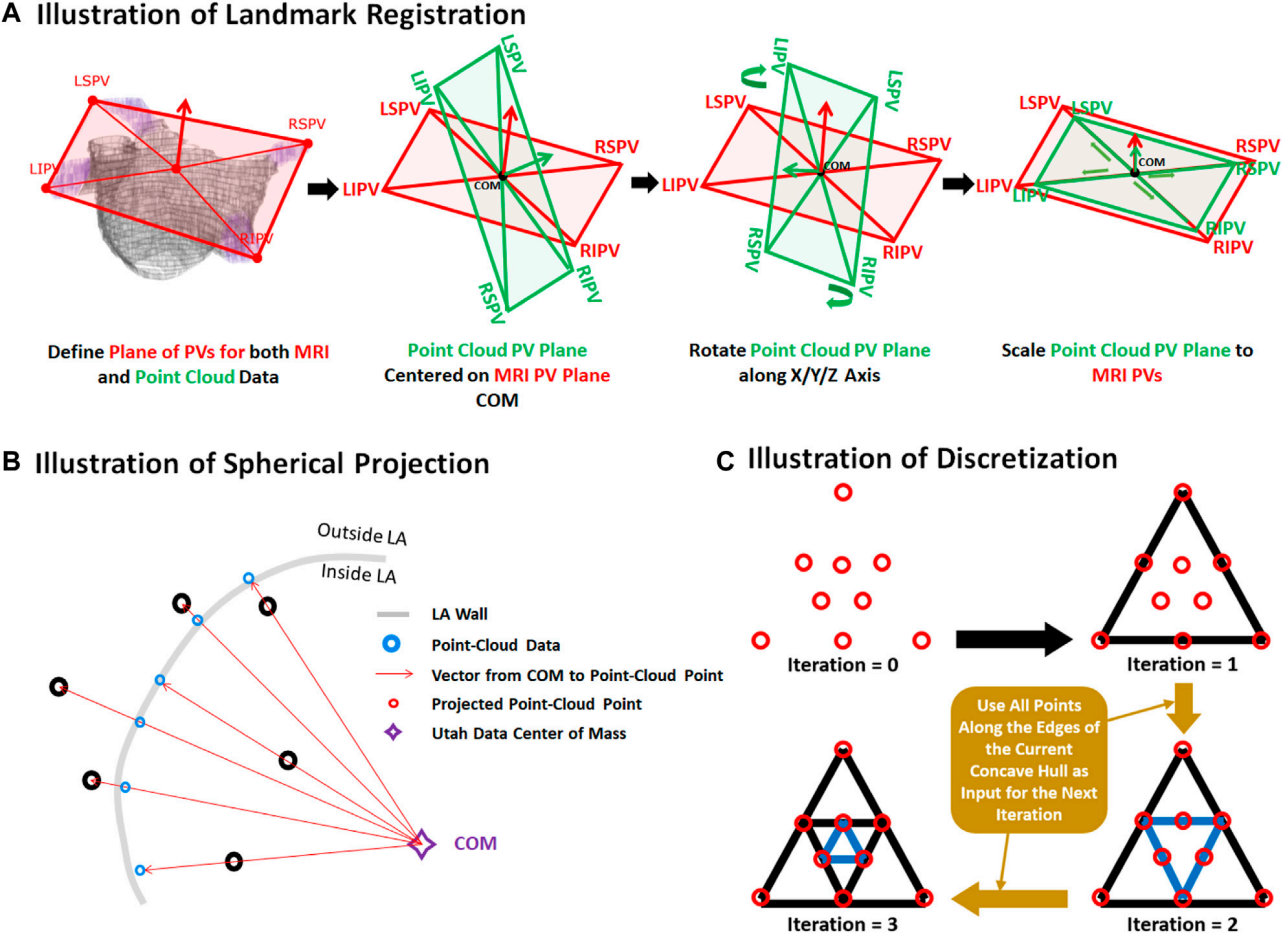
Illustration of the **(A)** registration, **(B)** projection, and **(C)** discretization stages for data generation from pairs of 3D left atrial (LA) geometry segmented from magnetic resonance imaging (MRI) and point clouds of the LA recorded during clinical mapping. Landmark registration was first performed to approximately match the pulmonary veins (PV) of the two LA geometries. This was performed by centering the center of mass (COM) of the point cloud PVs to the MRI. The point cloud was then rotated such that the PVs was able to closely match that of the MRI. The point cloud was lastly scaled for further refinement. The registered point cloud was spherically projected radially from the COM to the LA wall of the MRI to simulate a surface-point cloud recording on the MRI. The point cloud was lastly converted into a dense mesh using the concave hull.

The two datasets were merged by transforming the point cloud data using three stages: registration, projection, and discretization. For illustrative purposes, the three stages of the data generation process have been further outlined in Figure 2. As the coordinates of the PVs were labelled in both datasets, they were first used to register the point cloud through a series of translational, rotational and scaling matrix operations, obtaining the closest possible match of the landmarks. Since the aim of this initial step was to generate an approximate match between the two geometries, only rigid registration was performed. The registered point cloud was then spherically projected onto the surface of the 3D LA geometry using its center-of-mass as a reference point to produce an exact match between the two geometries. Finally, the projected point cloud was discretized using the alpha-concave hull algorithm (Foo et al., 2020) to generate a dense mesh of the point cloud. An alpha value of 5 was manually selected to produce an output which maintained the natural curvature of the LA. The concave hull algorithm was then iteratively applied three times such that in each iteration, points along all edges of the generated concave hull were added to the point cloud and inputted into the next iteration. This resulted in an exponential increase in the number of points after each iteration, transforming a point cloud vector of ∼4,000 samples to over 250,000 samples. Ultimately, this produced a dense mesh which was then discretized into integers forming a 3D image representing a partial shell of the LA.

The *paired training* dataset was split into training (N = 1,000), validation (N = 240), and testing (N = 300). The input data and labels were the point clouds and the LA segmented from the MRI dataset, respectively. The data was split such that an LA geometry from a given MRI was only present in one of the three datasets to avoid repeating labels.

#### 3.1.2 Test #1: Clinically Paired MRI and Point Cloud Data

MRIs with a resolution of 0.625 mm × 0.625 mm × 0.625 mm were acquired from 4 patients at Waikato Hospital, New Zealand, undergoing catheter ablation with the CARTO 3 mapping system (Prabhu et al., 2018). The average number of points recorded for the patients was 2,230 ± 790. Prior to the ablation procedure, the corresponding MRI scans were manually annotated by a team of experts to define the LA geometries. During clinical assessment, the LA was merged with the point clouds recorded during ablation mapping to spatially match the two data. For pre-processing, the point clouds were discretized using the method described above to create a 3D input LA shell for the CNN. The corresponding LA geometries from the MRIs were used as the ground truth for evaluation.

#### 3.1.3 Test #2: Clinically Paired CT and Point Cloud Data

CTs were obtained from 2 patients at The Royal Hospital Melbourne, Australia, undergoing catheter ablation with the EnsiteNavX mapping system (Njoku et al., 2018). The average number of mapped points was 2,818 ± 206. Similar to the *test #1* dataset above, the LA were manually segmented from the CTs and merged in the clinic with the point clouds. The point clouds were then discretized to create a 3D input LA shell for the CNN, and the respective LA geometries from the CTs were used as the ground truth for evaluation.

### 3.2 Evaluation

Several evaluation metrics were used to determine the accuracy of the CNN predictions. Evaluation was performed on all three of the paired training, *test #1*, and *test #2* datasets. The technical analysis included the dice score, surface-to-surface distance (STSD), sensitivity, and specificity. The dice score was defined similarly to the loss function in Eq. 1. STSD between the prediction, *A*, and ground truth, *B*, was defined as

$$STSD\left(A,B\right)=\frac{1}{n_{p}+n_{g}}\left(\sum_{p\prime =1}^{n_{p}}\sqrt{p\prime -p}+\sum_{g\prime =1}^{n_{g}}\sqrt{g\prime -g}\right)$$ (2)

where *a* and *b* are all the pixels locations within *A* and *B*, *n*_*A*_ is the number of pixels in *A*, *n*_*B*_ is the number of pixels in *B.* The sensitivity was defined as the number of true positives divided by the sum of the number of true positives and false negatives. The specificity was defined as the number of true negatives divided by the sum of the number of true negatives and false positives.

To measure the biological accuracy of the CNN predictions, we used the error in the LA diameter and volume. These are important biomarkers which have been shown to provide reliable information during clinical diagnosis and treatment stratification of atrial fibrillation (Zhuang et al., 2011; Njoku et al., 2018; Chen et al., 2022). The LA diameter was defined as

$${Ø}_{LA}\left(M\right)=\underset{i\in I}{\max}\left(\overset{J}{\underset{j=1}{\sum }}M_{ij}\right)$$ (3)

for a 2D slice of the 3D LA geometry with the maximum 2D width to obtain the overall maximum LA diameter, *M*, with dimensions *I* × *J*, where *J* was the anterior-posterior axis of the LA chamber. The atrial volume, *V*_*LA*_, was calculated by

$$V_{LA}\left(M\right)=\overset{X}{\underset{i=1}{\sum }}\overset{Y}{\underset{j=1}{\sum }}\overset{Z}{\underset{k=1}{\sum }}M_{ijk}\;$$ (4)

for a 3D mask, *M*, with dimensions *X × Y× Z*. The diameter and volume errors were then calculated by simply comparing the measures in the predictions with those from the ground truths. We also evaluated the coverage of the point cloud in the LA to measure its impact on the technical and biological accuracies. This was computed by

$$Coverage=\frac{\sum_{i}^{n}PT_{i}}{\sum_{j}^{m}LA_{surface}}$$ (5)

given the point cloud, *PT*, with a length of *n*, and the outer surface of the LA, *LA*_*surface*_, with *m* pixels, and *n < m*.

## 4 Results

### 4.1 Accuracy for Predicting the LA From Point Clouds

Tables 3, 4 show the complete evaluation results for the 3D LA reconstruction from point clouds in generated *paired training* dataset, and clinical *test #1* and *test #2* datasets. Overall, the proposed CNN achieved excellent accuracies for LA prediction, with dice scores of 93.2% for the *paired training* set, 92.4% for the *test #1* set, and 93.4% for the *test #2* set. These high accuracies showed that the CNN was able to successfully reconstruct the LA from the sparse inputs provided. The relatively low standard deviation of 2.3% on the 300 testing samples in the *paired training* set showed that the predictions were also very consistent. This was particularly seen in the two *test* sets with standard deviations of below 1% for the dice score. The CNN achieved an STSD of 1.1 pixels on the *paired training* set, and a more impressive 0.8 and 0.7 pixels on the *test #1* and *test #2* sets, showing the predicted LA was on average within 1 pixel of the ground truth. The high sensitivity of above 90% and the specificities of 99% showed that the CNN was able to distinguish between the positive and negative pixels with high certainties. Surprisingly, the approximately 4% higher sensitivity on the two clinical test sets indicated the CNN was able to capture the LA pixels much more effectively than in the *paired training* set.

**TABLE 3:** Technical evaluation for left atrium reconstruction from point clouds in the 300 generated (*Paired training*), 4 clinical MRI (*Test #1*), and 2 clinical CT (*Test #2*) data.

Dataset	Dice	STSD	Sensitivity	Specificity
*Paired training data*	93.2 ± 2.3%	1.16 ± 0.48px	90.6 ± 3.7%	99.7 ± 0.1%
*Test#1*	92.4 ± 0.8%	0.76 ± 0.05px	94.9 ± 0.6%	99.2 ± 0.2%
*Test#2*	93.4 ± 0.6%	0.66 ± 0.05px	95.0 ± 0.3%	99.2 ± 0.1%

CT, computed tomography; MRI, magnetic resonance imaging.

**TABLE 4:** Biological evaluation for left atrium reconstruction from point clouds in the 300 generated (*Paired training*), 4 clinical MRI (*Test #1*), and 2 clinical CT (*Test #2*) data.

Dataset	Diameter error	Volume error
*Paired training data*	4.4 ± 5.2%	5.9 ± 4.1%
*Test#1*	2.6 ± 1.2%	5.2 ± 1.0%
*Test#2*	3.0 ± 1.0%	3.3 ± 1.9%

CT, computed tomography; MRI, magnetic resonance imaging.

The predicted LA were also biologically accurate on average, obtaining low diameter and volume errors of 4.4% and 5.9%, respectively (Table 4). The higher sensitivities in the two *test* sets also resulted in lower diameter and volume errors with 2.6% and 3.0% errors for the diameter, and 5.2% and 3.3% for the volume in the *test #1* and *test #2* sets, respectively. We also compared the biological measurements between the ground truth and predicted LA to determine the error source. We found that the mean predicted diameter of 39.9 mm and volume of 49.0 cm^3^ were lower when compared to the 41.5 mm and 52.4 cm^3^ ground truth measurements. This revealed the CNN had a tendency to slightly underestimate the LA when analyzing point clouds.

### 4.2 Visualization and Error Analysis

**FIGURE 3:**
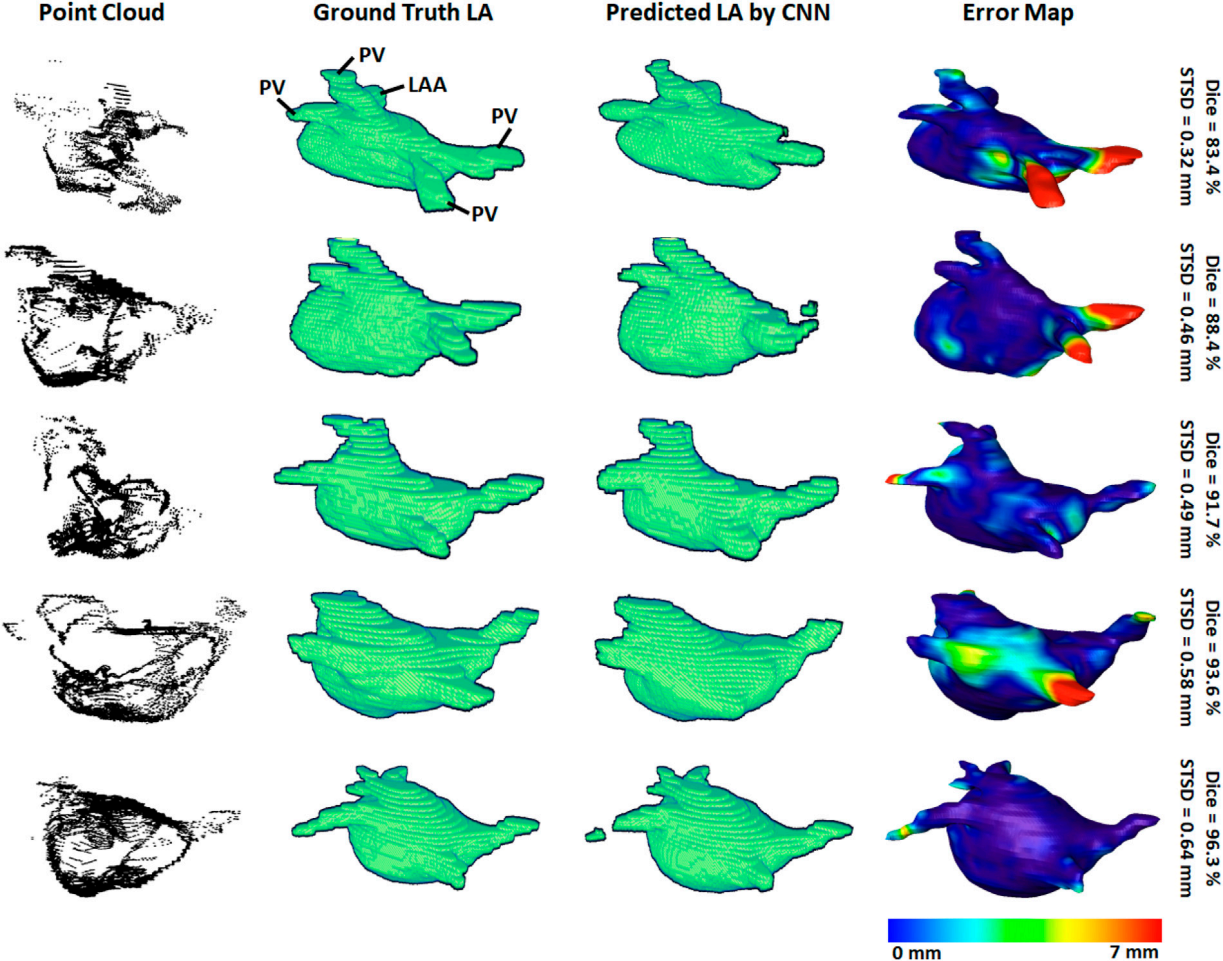
3D visualizations of the left atrial (LA) reconstructions of five samples in the paired training dataset. The reconstructions with the highest dice scores are in the bottom row and the top row contains the reconstructions with the lowest dice scores. The point-clouds inputs are shown in the first column. The ground truths obtained by manually segmenting the LGE-MRIs are shown in the second column. The reconstructions predicted by the convolutional neural network (CNN) are shown in the third column. The surface-to-surface distance (STSD) error maps between the ground truths and the predictions are shown in the fourth column, with the colors being normalized between 0 and 7 mm for the five samples. LAA, left atrial appendage; PV, pulmonary vein.

3D visualizations of the ground truth and predictions produced by the CNN were produced for further error analysis. Figure 3 shows five samples of predictions made by the CNN in order of increasing accuracy, representing the range of accuracies obtained in the *paired training* set. The input point cloud was also shown with the corresponding ground truth LA geometry. From the samples shown, it was observed that the degree of coverage depicted by the input data had a significant impact on the accuracy of prediction. This was clearly visible in the first row where the input point cloud had low coverage. The CNN was therefore forced to generate many anatomical features without guidance, based only on the shape of the existing input. The fifth row showed an input containing extremely good coverages, naturally making the prediction much more accurate. However, rows one to four also revealed the power of the CNN for data generation, as the outputs, regardless of dice score, were all anatomically similar to the ground truths. This also showed that the CNN would be effective on clinically recorded point clouds which do not fully cover the entire LA surface. Expectedly, the most erroneous regions were the PVs when a distance-error map was computed between the predictions and ground truths. This was due to the PVs having a thin and inconsistent shape compared to the rest of the LA, creating difficulties for the CNN to consistently define.

**FIGURE 4:**
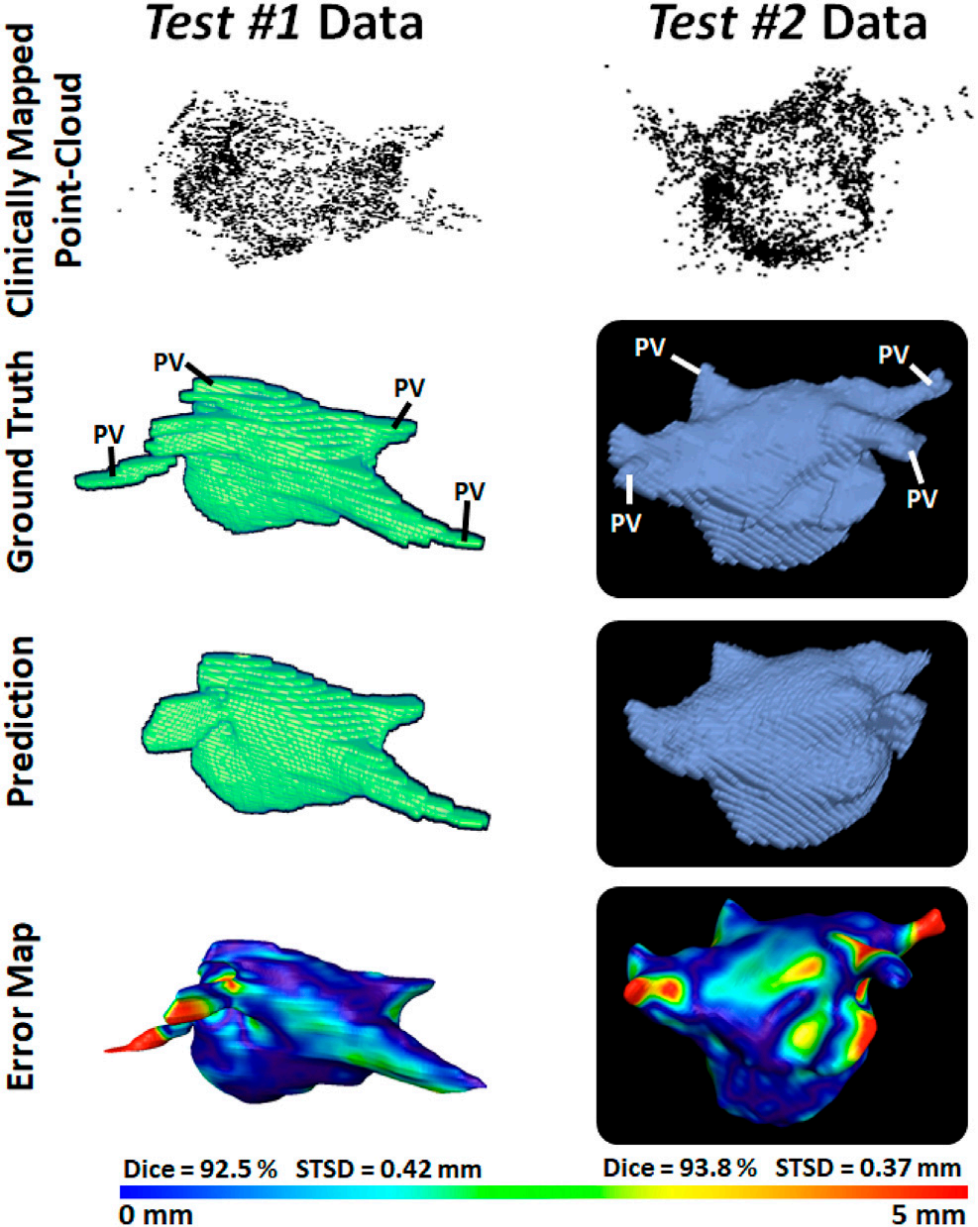
3D Visualizations of the left atrial (LA) reconstruction for one sample each from test #1 (left column) and test #2 (right column) clinical datasets. The point cloud recorded with the commercial mapping systems are shown in the first row, along with the LA geometry obtained from segmenting magnetic resonance imaging (MRI) and computed tomography (CT) in the second row. The predicted LA are shown in the third row, and the surface-to-surface distance (STSD) error maps between the ground truths and the predictions are shown in the fourth row. The individual dice and STSD scores are shown for each sample. PV, pulmonary vein.

To demonstrate our method is adaptable and feasible on the two real clinical datasets (*test #1* and *test #2*). we displayed the prediction and ground truth of one sample from each dataset in Figure 4. In general, it can be seen the point cloud in these datasets covered a significantly larger proportion of the LA compared to the *paired training* dataset. This led to the CNN performing better given the more complete LA shells which were generated from the point clouds. Furthermore, the adaptability of our CNN can be seen in the results for the *test #2* data. The LA was acquired from CTs, as opposed to MRIs which were used in both the *paired training* and *test #1* datasets, leading to a significantly different geometry. Nevertheless, our CNN effectively predicted the CT geometry although it was only trained on MRIs geometries, showing our approach was independent of the mapping system and image modality.

### 4.3 Impact of Point Cloud Coverage on the Accuracy

We analyzed the impact of the coverage of the point cloud over the target output LA on the evaluation scores obtained in our results (Table 5). The average coverage across the *paired training* dataset was 30%, while the *test #1* and *test #2* sets had coverages of 40% and 44%, respectively. The standard deviation of the coverage on the *paired training* set was 5.4% and contained a range of 19%–40%. This indicated there was a wide range of point cloud coverages for the CNN during training, allowing it to be applicable to a range of distributions during prediction. Interestingly, the mean coverages of the two *test* sets were above and outside the range of the *paired training* set, showing the point clouds acquired in the clinical sets were of higher quality. This was a potential explanation for the increased sensitivities on the two clinical sets, as the higher coverage allowed the CNN to predict the entire LA geometry with slightly greater precision compared to the training set. Although this did not result in an increased dice score as the specificities of the two clinical datasets were lower compared to that of the training set. This was also visible in Figures 3 vs. Figure 4 which showed a smoother point cloud distribution for the *test #1* and *test #2* data. The 5% higher coverage in *test #2* compared to *test #1* was also a potential reason for the 1% higher dice score between the two clinical testing sets.

**TABLE 5:** The point cloud coverage over the left atrium for the generated (*Paired training*), clinical MRI (*Test #1*), and clinical CT (*Test #2*) datasets.

Dataset	Coverage
*Paired training data*	30.3 ± 5.4%
*Test#1*	39.5 ± 1.5%
*Test#2*	44.4 ± 2.6%

CT, computed tomography; MRI, magnetic resonance imaging.

**FIGURE 5:**
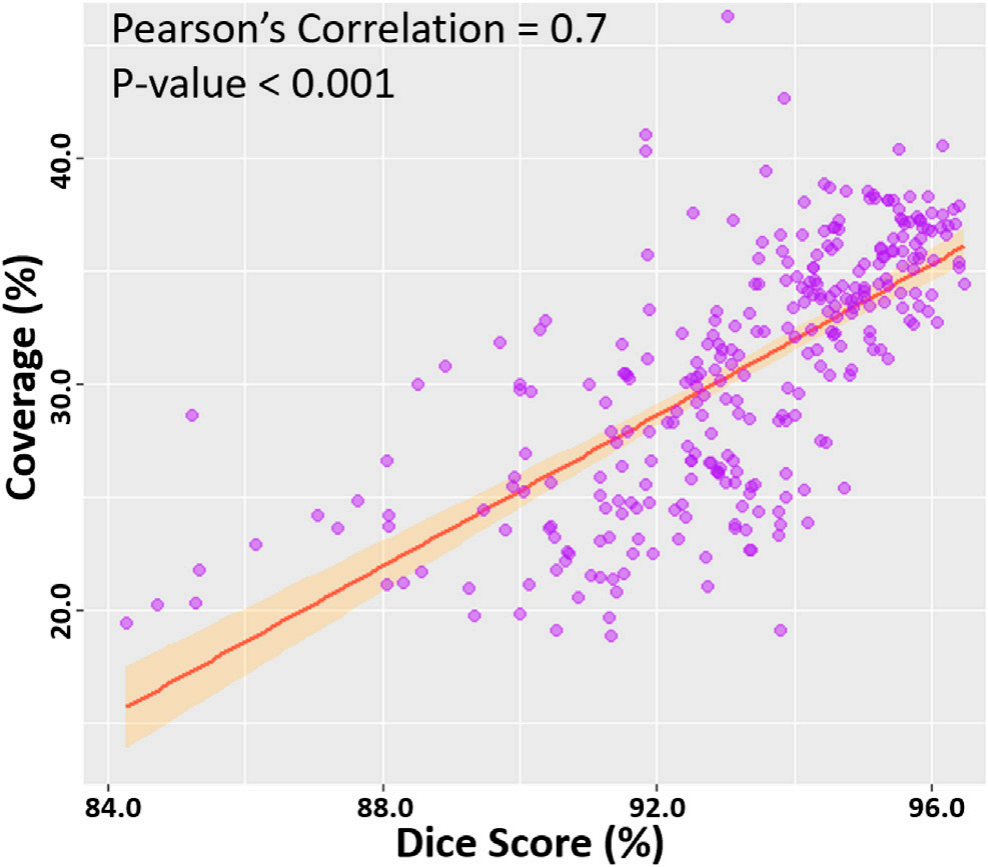
Correlation of the point cloud coverage with the dice score in the testing datasets. The line of best fit is shown, along with the Pearson’s correlation value and *p*-value. The band shows the standard deviation of the points along the line.

We then computed the Pearson’s correlation between the point cloud coverage in all data and the accuracies obtained by our CNN (Figure 5). Overall, the coverage was significantly and strongly correlated to both the dice score and sensitivity, with correlations of 0.7. This was a potential explanation for the increased sensitivity on the two clinical sets, as the higher coverage allowed the CNN to predict the entire LA geometry with greater precision. The coverage was also moderately correlated to the STSD with a value of 0.6 and statistical significance. Expectedly, the errors for the diameter and volume were both negatively correlated with the coverage, as higher coverages resulted in better predictions of the biological measurements, and thus lower errors. While the diameter error had a low correlation of −0.1, the volume error had a moderate negative correlation of −0.6. This was due to the diameter only being measured in one dimension, and thus being impacted less by the overall LA reconstruction accuracy, while the volume was influenced by all three dimensions.

## 5 Discussion

Direct surface reconstruction of organs, such as the LA, from point clouds is a challenging task. Prevailing methods of analysis primarily focus on the application of CNNs for the classification and segmentation of point cloud representations of 3D objects or scenery. Well-established research into the reconstruction of 3D surface geometries directly from sparse inputs such as point cloud is therefore limited. Furthermore, the current commercial software used to perform clinical mapping and the subsequent LA reconstruction from the point clouds recorded is inefficient by requiring additional imaging prior to the procedure. The efficacy of the proprietary software also remains difficult to validate, and open research in the area is lacking.

To address the current issues, our study is one of the first to propose a fully automated framework for the reconstruction of the LA geometry directly from point clouds. Our study is also one of few to develop a CNN for the surface reconstruction of 3D geometries given a set of partially complete information such as the sparse point clouds data described. Overall, the proposed CNN produced LA predictions with high-performance accuracies across multiple metrics for both technical and biological evaluation. The CNN obtained dice scores surpassing a prior study which investigated a similar task with over 7% accuracy improvements (Baram et al., 2018). The low surface-to-surface distance, LA diameter, and LA volume errors showed our approach produced anatomically accurate predictions, which is a highly important feature for clinical applications. The clinical applicability of our approach was further demonstrated on the two clinical point cloud datasets acquired with the most commonly used CARTO and EnSiteNavX mapping systems. Experimental results showed the CNN achieved similarly accurate and consistent predictions when compared to LA geometries segmented from the MRIs and CTs in the clinical datasets. By conducting the first study which utilized real patient data for both training and testing, this study would ideally establish a solid benchmark in this under-investigated field.

An important component of CNN pipelines for point cloud analysis involves the pre-processing of the point clouds data into fixed-sized inputs. Similar to prior studies, we retained the original dimensionality and important spatial information of the inputs by directly discretizing the 3D point cloud into an image volume (Tchapmi et al., 2017). However, the pre-processing step in our study was significantly enhanced by the proposed iterative concave-hull algorithm, which exponentially increased the number of data points with low computational costs. The increased number of points resulted in smooth image volumes after discretization. This was an improvement on past methods which attempted to directly discretize low-density point clouds to produce sparse images which were difficult and computationally expensive to analyze by the CNN. As the pre-processed volumes contained a high density of information, this also benefited the performance of the CNN by providing concentrated data with a relatively low memory cost, leading to more precise predictions with greater efficiency. A further step for ensuring effective feature learning on the pre-processed point clouds involved the utilization of larger convolutional kernels to increase the receptive field of the CNN. The CNN was also enhanced with the use of feature forwarding connections, allowing it to retain and combine features at multiple receptive levels, maximizing the information extracted from the relatively sparse input information provided. Due to the high class-imbalance of the point clouds which often induces CNNs to produce completely empty predictions, we implemented a dice loss to prioritize non-background pixels. Residual blocks and batch normalization were also included to increase the ease of convergence and decrease the likelihood that the parameter optimization process does not stall at an undesirable local minimum during training.

Our study contains several limitations, which can potentially be addressed in future studies. Experiments on our CNN showed that although it performed excellently overall, its accuracy was directly dependent on the coverage of the point cloud. While most clinical point cloud recordings nowadays maintain good coverage over the entire LA chamber as seen in the samples in this study, future methods should specifically be aimed to address low coverage maps. Such methods could involve statistical shape models which artificially enhance the coverage by using aggregated anatomical features from past data to estimate the location of potential landmarks. Future research should also investigate changes to the CNN architecture to improve its accuracy in general, such as introducing adversarial pathways or auxiliary outputs which are commonly used for image reconstruction. The loss function could be improved by introducing anatomical constraints to ensure the outputs contain all key anatomical landmarks which would be very beneficial in clinical applications. Methods for directly analysing point clouds would also be explored in future studies including graph convolutional networks which would save computational time during the data preparation and remove the need for the points to be converted into image volumes. Direct learning on the point cloud data may also decrease potential biases introduced during the current discretization step, as well as provide more flexibility when handling different datasets in the future. Such methods may also be used in conjunction with our current pipeline as an additional pathway to further strengthen our approach. Finally, future studies should ideally utilize larger samples of clinical data through more extensive collaborations with international clinical centers to further validate the robustness of the framework. Such clinical trials would ideally involve both LGE-MRI scanning and anatomical mapping in every patient, with further processing using EnsiteNavX or CARTO 3 to merge and match the geometries of the atrium in both acquisitions. Generative neural networks could also potentially mitigate these issues by allowing semi-supervised learning on unlabeled datasets which are more widely available (Chen et al., 2021) and providing greater learning capacities when training on limited labelled data [36].

## 6 Conclusion

In this study, we have developed and evaluated a 3D CNN for robust automatic LA reconstruction from point clouds recorded with clinical mapping during ablation. Our algorithm enables the reconstruction of the LA in 3D with a dice accuracy of 93%, STSD of approximately 1 pixel, and accurate estimations of clinical measures. The framework was further tested on two independent cross-modality clinical datasets, and produced similarly impressive evaluation results. Our study may lead to the development of a more accurate and efficient real-time LA reconstruction approach, which can potentially be used to improve clinical guidance during ablation procedures for the treatment of cardiac diseases.

## Data Availability

The data analyzed in this study is subject to the following licenses/restrictions: It is clinical data from other centres. Requests to access these datasets should be directed to JZ, j.zhao@auckland.ac.nz.
